# Retraction: Efficient lung cancer detection using computational intelligence and ensemble learning

**DOI:** 10.1371/journal.pone.0324356

**Published:** 2025-05-12

**Authors:** The PLOS One Editors

The *PLOS One* Editors retract this article [1] due to concerns about reliance on retracted work cited in References 18, 20, 22, 25, 28, 32, and 33.

RJ, PS, and WB did not agree with the retraction. MA either did not respond directly or could not be reached.
